# Supplementary material for: RNA-Sequencing, Physiological and RNAi Analyses Provide Insights into the Response Mechanism of the ABC-Mediated Resistance to Verticillium dahliae Infection in Cotton
Source: Genes (Basel). 2019 Feb 1;10(2):110. doi: 10.3390/genes10020110 (PMC6410047; doi:10.3390/genes10020110)
Supplement: Supplementary file 1 [file genes-10-00110-s001.zip › Supplementary files/Supplementary Table 1 Hisat was used to compare the valid pre-processed data (Valid Data) with the reference genome, and Reads was compared with the reference.docx]

| Sample | Valid reads | Mapped reads | Mapped reads | Unique Mapped reads | Unique Mpped reads | Multi Mapped reads | Multi Mapped reads | PE Mappe reads | PE Mappe reads |
| --- | --- | --- | --- | --- | --- | --- | --- | --- | --- |
| Gr_0L | 45174949 | 41088840 | 91.13% | 31891244 | 70.87% | 9197596 | 20.26% | 38381750 | 85.18% |
| Gr_12L | 43550318 | 41141907 | 94.47% | 32569572 | 74.83% | 8572334 | 19.64% | 39136393 | 89.84% |
| Gr_48L | 40872607 | 38946472 | 95.29% | 31057088 | 75.98% | 7889384 | 19.31% | 37096265 | 90.77% |
| Gr_0S | 44021567 | 42146359 | 95.74% | 32587854 | 74.06% | 9558506 | 21.69% | 40389025 | 91.74% |
| Gr_12S | 45813186 | 43789549 | 95.58% | 33932948 | 74.09% | 9856602 | 21.49% | 41914190 | 91.49% |
| Gr_48S | 42853911 | 41065220 | 95.84% | 31998937 | 74.72% | 9066283 | 21.12% | 39319607 | 91.77% |
| Gr_0R | 45704989 | 43682019 | 95.58% | 33467358 | 73.23% | 10214661 | 22.35% | 41906290 | 91.70% |
| Gr_12R | 46111933 | 43982376 | 95.38% | 33656289 | 73.02% | 10326087 | 22.37% | 42037968 | 91.17% |
| Gr_48R | 43866601 | 41968459 | 95.70% | 32554678 | 74.28% | 9413781 | 21.42% | 40173806 | 91.63% |
| Gtr_0L | 43769161 | 38829808 | 88.73% | 23209491 | 53.03% | 15620317 | 35.70% | 35847235 | 81.93% |
| Gtr_12L | 41421274 | 36627395 | 88.39% | 21711273 | 52.38% | 14916123 | 36.01% | 33745271 | 81.41% |
| Gtr_48L | 45889514 | 40644443 | 88.59% | 24041529 | 52.41% | 16602914 | 36.19% | 37402977 | 81.55% |
| Gtr_0S | 43840067 | 38628705 | 88.17% | 22689929 | 51.80% | 15938775 | 36.37% | 35260481 | 80.53% |
| Gtr_12S | 43571182 | 38419112 | 88.24% | 22621675 | 52.00% | 15797437 | 36.24% | 35089705 | 80.63% |
| Gtr_48S | 48625743 | 41811833 | 86.29% | 24636646 | 50.94% | 17175187 | 35.35% | 37583611 | 77.64% |
| Gtr_0R | 46138478 | 40332508 | 87.33% | 23927011 | 51.81% | 16405497 | 35.52% | 36586750 | 79.17% |
| Gtr_12R | 42689842 | 37994822 | 89.00% | 22581963 | 52.89% | 15412859 | 36.12% | 34722870 | 81.34% |
| Gtr_48R | 42373817 | 37487813 | 88.41% | 22092617 | 52.10% | 15395196 | 36.31% | 34210921 | 80.64% |
| Gth_0L | 39685912 | 35354030 | 89.07% | 20648174 | 52.03% | 14705855 | 37.04% | 32710146 | 82.40% |
| Gth_12L | 45436039 | 40197818 | 88.50% | 23824669 | 52.43% | 16373149 | 36.07% | 37507421 | 82.56% |
| Gth_48L | 41902559 | 37087854 | 88.57% | 21753894 | 51.96% | 15333960 | 36.61% | 34533379 | 82.47% |
| Gth_0S | 43353597 | 38327365 | 88.39% | 22798365 | 52.59% | 15529000 | 35.81% | 34910708 | 80.50% |
| Gth_12S | 46946611 | 41013849 | 87.42% | 24355821 | 51.94% | 16658028 | 35.48% | 36844071 | 78.54% |
| Gth_48S | 42695944 | 37444405 | 87.69% | 22389213 | 52.42% | 15055191 | 35.28% | 33832392 | 79.25% |
| Gth_0R | 44295005 | 39256626 | 88.61% | 23278745 | 52.54% | 15977880 | 36.07% | 35684011 | 80.53% |
| Gth_12R | 51588587 | 45593430 | 88.36% | 27051173 | 52.42% | 18542257 | 35.94% | 41282671 | 80.01% |
| Gth_48R | 47914659 | 42392977 | 88.48% | 25373749 | 52.95% | 17019228 | 35.53% | 38254203 | 79.87% |

**Supplementary Table 1.** Hisat was used to compare the valid pre-processed data (Valid Data) with the reference genome, and Reads was compared with the reference genome according to the location information of the gene specified in the genome annotation file.
