# Supplementary material for: RNA-Sequencing, Physiological and RNAi Analyses Provide Insights into the Response Mechanism of the ABC-Mediated Resistance to Verticillium dahliae Infection in Cotton
Source: Genes (Basel). 2019 Feb 1;10(2):110. doi: 10.3390/genes10020110 (PMC6410047; doi:10.3390/genes10020110)
Supplement: Supplementary file 1 [file genes-10-00110-s001.zip › Supplementary files/Supplementary Table 2. Physiochemical properties of the ABC genes in Gossypium raimondii..docx]

**Supplementary Table 2.** Physiochemical properties of the ABC genes in *Gossypium raimondii.*

| Gene ID | Length (bp) | Transcript Length (bp) | CDS Length (bp) | CDS GC Content (%) | Exon Number | Mean Exon Length (bp) | Mean Intron Length (bp) | Protein Length (aa) | Molecular Weight (kDa) | Charge | Isoelectric Point | Grand Average of Hydropathy |
| --- | --- | --- | --- | --- | --- | --- | --- | --- | --- | --- | --- | --- |
| Gorai.001G003100 | 7,414 | 4,194 | 4,056 | 40.6 | 19 | 220.7 | 178.9 | 1,351 | 153.047 | 21.5 | 8.547 | 0.048 |
| Gorai.001G057200 | 7,520 | 4,544 | 4,389 | 41.2 | 24 | 189.3 | 129.4 | 1,462 | 164.6 | 7 | 7.107 | 0.078 |
| Gorai.001G057400 | 7,549 | 4,630 | 4,473 | 40.9 | 23 | 201.3 | 132.7 | 1,490 | 168.048 | 6 | 6.963 | 0.088 |
| Gorai.001G068300 | 5,942 | 2,524 | 2,109 | 42.7 | 10 | 252.4 | 379.2 | 702 | 78.405 | 15.5 | 9.294 | 0.097 |
| Gorai.001G075900 | 4,947 | 2,385 | 1,887 | 43.9 | 17 | 140.3 | 160.1 | 628 | 67.873 | 13 | 9.338 | 0.153 |
| Gorai.001G115700 | 34,157 | 4,895 | 4,302 | 41.6 | 24 | 204 | 1,272.30 | 1,433 | 161.015 | 19 | 8.473 | 0.039 |
| Gorai.001G116000 | 11,221 | 4,639 | 4,317 | 42 | 24 | 193.3 | 286.2 | 1,438 | 160.685 | 19.5 | 8.748 | 0.085 |
| Gorai.001G133500 | 4,249 | 2,881 | 2,151 | 44.5 | 1 | 1,440.50 | No intron | 716 | 79.888 | -3 | 6.209 | -0.609 |
| Gorai.001G147100 | 11,043 | 4,618 | 4,242 | 40.4 | 23 | 200.8 | 292 | 1,413 | 161.286 | 33 | 8.759 | 0.05 |
| Gorai.001G147200 | 4,467 | 2,189 | 2,166 | 40.7 | 14 | 156.4 | 175.2 | 721 | 82.224 | 8.5 | 7.858 | -0.004 |
| Gorai.001G182400 | 9,592 | 5,495 | 4,623 | 42.3 | 13 | 422.7 | 336.3 | 1,540 | 170.615 | 13 | 7.565 | 0.252 |
| Gorai.001G249000 | 2,259 | 2,259 | 2,058 | 45.1 | 1 | 2,259.00 | No intron | 685 | 75.722 | 17 | 9.401 | 0.178 |
| Gorai.001G254500 | 2,460 | 1,413 | 990 | 43.2 | 5 | 282.6 | 191.8 | 329 | 37.076 | 6 | 7.543 | -0.21 |
| Gorai.001G256100 | 6,538 | 4,353 | 3,750 | 44.5 | 10 | 435.3 | 242.8 | 1,249 | 136.101 | 11 | 7.708 | 0.122 |
| Gorai.001G258400 | 5,879 | 4,201 | 3,783 | 43.7 | 7 | 600.1 | 279.7 | 1,260 | 137.483 | 15 | 8.156 | 0.085 |
| Gorai.001G258500 | 4,598 | 3,990 | 3,801 | 41 | 7 | 570 | 101.3 | 1,266 | 137.88 | 19.5 | 8.72 | 0.117 |
| Gorai.002G023500 | 6,832 | 2,838 | 2,256 | 42.1 | 9 | 315.3 | 499.3 | 751 | 84.572 | 3.5 | 7.004 | -0.154 |
| Gorai.002G057800 | 12,432 | 4,601 | 4,374 | 41.8 | 24 | 191.7 | 340.5 | 1,457 | 163.69 | 2 | 6.687 | 0.092 |
| Gorai.002G062500 | 1,971 | 1,971 | 1,830 | 42.8 | 1 | 1,971.00 | No intron | 609 | 68.201 | 18 | 9.385 | 0.145 |
| Gorai.002G073600 | 2,570 | 2,315 | 1,884 | 42.6 | 4 | 578.8 | 85 | 627 | 69.419 | 10.5 | 8.744 | 0.146 |
| Gorai.002G146400 | 23,054 | 6,284 | 5,673 | 40.3 | 40 | 157.1 | 430 | 1,890 | 210.467 | 19 | 7.524 | 0.14 |
| Gorai.002G153900 | 6,941 | 2,843 | 2,256 | 46.2 | 10 | 284.3 | 416.3 | 751 | 83.206 | 11 | 8.715 | 0.011 |
| Gorai.002G162300 | 6,289 | 3,246 | 3,246 | 41.6 | 14 | 231.9 | 234.1 | 1,081 | 118.917 | 42.5 | 9.379 | -0.132 |
| Gorai.002G180700 | 8,007 | 2,760 | 2,163 | 44.8 | 12 | 230 | 477 | 720 | 78.494 | 12 | 8.843 | 0.025 |
| Gorai.002G186500 | 8,476 | 2,926 | 2,178 | 42.8 | 18 | 162.6 | 326.3 | 725 | 81.57 | 22 | 8.989 | 0.099 |
| Gorai.002G188900 | 4,369 | 3,873 | 3,753 | 43.5 | 6 | 645.5 | 99.2 | 1,250 | 136.968 | 35 | 9.591 | 0.028 |
| Gorai.002G233500 | 5,881 | 2,837 | 2,247 | 43.6 | 9 | 315.2 | 380.5 | 748 | 83.039 | 17.5 | 9.286 | -0.013 |
| Gorai.002G246800 | 6,029 | 4,665 | 3,987 | 43.3 | 10 | 466.5 | 151.6 | 1,328 | 145.57 | 13 | 7.631 | 0.017 |
| Gorai.002G252600 | 2,132 | 2,132 | 2,091 | 42.3 | 1 | 2,132.00 | No intron | 696 | 77.459 | 22 | 10.045 | 0.136 |
| Gorai.003G038900 | 10,691 | 4,231 | 4,023 | 41.7 | 24 | 176.3 | 280.9 | 1,340 | 150.367 | 38 | 9.407 | -0.07 |
| Gorai.003G047600 | 4,625 | 2,625 | 2,073 | 43.6 | 9 | 291.7 | 250 | 690 | 78.35 | 5 | 7.963 | -0.59 |
| Gorai.003G062100 | 8,911 | 4,729 | 4,479 | 43.3 | 21 | 225.2 | 209.1 | 1,492 | 168.737 | 14 | 8.26 | 0.025 |
| Gorai.003G064800 | 13,039 | 4,408 | 4,332 | 41 | 24 | 183.7 | 375.3 | 1,443 | 162.755 | 20.5 | 8.218 | 0.053 |
| Gorai.003G070300 | 2,306 | 2,306 | 1,809 | 43.9 | 1 | 2,306.00 | No intron | 602 | 66.833 | 18.5 | 9.375 | 0.361 |
| Gorai.003G082700 | 7,327 | 4,678 | 4,578 | 40.5 | 12 | 389.8 | 240.8 | 1,525 | 169.718 | 4 | 6.719 | 0.227 |
| Gorai.003G089800 | 3,927 | 2,907 | 2,118 | 41.9 | 11 | 264.3 | 102 | 705 | 78.59 | 13.5 | 8.799 | 0.114 |
| Gorai.003G144400 | 6,807 | 2,986 | 2,172 | 43.6 | 12 | 248.8 | 338.2 | 723 | 80.645 | 24 | 9.429 | 0.194 |
| Gorai.003G153100 | 11,340 | 3,156 | 2,220 | 43 | 19 | 166.1 | 454.7 | 739 | 81.481 | 21.5 | 9.682 | 0.017 |
| Gorai.003G183800 | 8,302 | 4,140 | 3,846 | 44.2 | 11 | 376.4 | 416.2 | 1,281 | 140.679 | 26 | 9.296 | 0.159 |
| Gorai.004G033800 | 3,080 | 2,322 | 1,893 | 41.8 | 9 | 258 | 94.8 | 630 | 71.228 | 10.5 | 8.774 | 0.017 |
| Gorai.004G089200 | 5,617 | 4,809 | 4,530 | 43.7 | 9 | 534.3 | 101 | 1,509 | 169.333 | 4.5 | 6.765 | 0.175 |
| Gorai.004G111000 | 7,150 | 4,689 | 4,353 | 41.9 | 24 | 195.4 | 107 | 1,450 | 163.775 | 17 | 8.39 | 0.072 |
| Gorai.004G111900 | 6,029 | 2,639 | 2,157 | 43.6 | 16 | 164.9 | 226 | 718 | 77.94 | 16.5 | 9.411 | 0.186 |
| Gorai.004G131200 | 8,190 | 2,833 | 2,151 | 44.2 | 18 | 157.4 | 315.1 | 716 | 79.877 | -9 | 5.801 | -0.306 |
| Gorai.004G138100 | 10,930 | 4,547 | 4,293 | 42.4 | 24 | 189.5 | 277.5 | 1,430 | 162.153 | 19 | 8.316 | 0.073 |
| Gorai.004G147200 | 6,095 | 4,374 | 3,876 | 44.9 | 13 | 336.5 | 143.4 | 1,291 | 139.317 | 0.5 | 6.577 | 0.13 |
| Gorai.004G177600 | 5,262 | 2,446 | 1,845 | 43 | 12 | 203.8 | 251 | 614 | 69.1 | 8 | 7.902 | -0.198 |
| Gorai.004G178100 | 3,673 | 2,537 | 1,962 | 48.1 | 5 | 507.4 | 284 | 653 | 72.778 | 17.5 | 9.036 | 0.124 |
| Gorai.004G261400 | 5,508 | 1,622 | 1,050 | 40.8 | 11 | 147.5 | 386.9 | 349 | 38.259 | 5.5 | 7.777 | -0.19 |
| Gorai.005G134100 | 3,762 | 2,937 | 2,118 | 46.1 | 9 | 326.3 | 103.1 | 705 | 78.531 | 13 | 8.997 | 0.118 |
| Gorai.005G172300 | 6,359 | 5,277 | 4,380 | 41.7 | 11 | 479.7 | 108.2 | 1,459 | 164.204 | 25.5 | 8.377 | 0.136 |
| Gorai.005G214200 | 4,690 | 3,899 | 3,792 | 42.6 | 10 | 389.9 | 87.9 | 1,263 | 139.312 | 9 | 7.316 | 0.12 |
| Gorai.005G221600 | 2,596 | 2,172 | 1,959 | 44.4 | 6 | 362 | 84.8 | 652 | 71.991 | 14.5 | 9.1 | 0.155 |
| Gorai.005G221800 | 1,286 | 918 | 918 | 43.9 | 6 | 153 | 73.6 | 305 | 32.895 | 8.5 | 9.168 | -0.21 |
| Gorai.005G221900 | 2,305 | 1,878 | 1,878 | 44.1 | 6 | 313 | 85.4 | 625 | 69.073 | 14 | 9.091 | 0.198 |
| Gorai.005G222100 | 2,860 | 2,393 | 2,046 | 44.5 | 6 | 398.8 | 93.4 | 681 | 75.118 | 13 | 8.558 | 0.072 |
| Gorai.005G222400 | 3,067 | 1,875 | 1,218 | 43.3 | 10 | 187.5 | 132.4 | 405 | 45.574 | 9 | 8.448 | 0.148 |
| Gorai.006G001900 | 2,036 | 2,036 | 1,812 | 40.3 | 1 | 2,036.00 | No intron | 603 | 68.168 | 13.5 | 8.455 | 0.205 |
| Gorai.006G021600 | 6,227 | 4,379 | 3,750 | 45 | 10 | 437.9 | 205.3 | 1,249 | 136.019 | 8.5 | 7.47 | 0.128 |
| Gorai.006G026800 | 4,394 | 3,768 | 3,768 | 41.2 | 7 | 538.3 | 104.3 | 1,255 | 137.388 | 14 | 7.745 | 0.123 |
| Gorai.006G033600 | 5,296 | 1,905 | 1,905 | 42.9 | 20 | 95.3 | 162.1 | 634 | 70.718 | 12 | 8.258 | 0.145 |
| Gorai.006G124200 | 2,315 | 2,315 | 1,980 | 43.2 | 1 | 2,315.00 | No intron | 659 | 74.406 | 8 | 7.782 | 0.115 |
| Gorai.006G124600 | 3,688 | 2,726 | 2,178 | 44 | 1 | 908.7 | No intron | 725 | 80.786 | -4.5 | 6.068 | -0.622 |
| Gorai.006G126900 | 6,777 | 2,871 | 2,091 | 43.5 | 9 | 319 | 488.3 | 696 | 77.189 | 17 | 9.187 | 0.099 |
| Gorai.006G130900 | 6,959 | 2,696 | 1,983 | 45.2 | 3 | 898.7 | 2,131.50 | 660 | 72.952 | 27 | 9.695 | 0.17 |
| Gorai.006G147000 | 6,235 | 2,996 | 2,259 | 46.4 | 11 | 272.4 | 323.9 | 752 | 83.339 | 16 | 9.367 | -0.039 |
| Gorai.006G157100 | 6,383 | 4,320 | 4,320 | 43.6 | 21 | 205.7 | 87.3 | 1,439 | 162.756 | 15 | 8.102 | 0.096 |
| Gorai.006G157200 | 2,971 | 2,243 | 2,136 | 43.7 | 9 | 249.2 | 91 | 711 | 80.817 | 6 | 7.165 | -0.292 |
| Gorai.006G163000 | 6,540 | 4,485 | 3,885 | 44.9 | 10 | 448.5 | 228.1 | 1,294 | 141.033 | 19.5 | 8.502 | 0.083 |
| Gorai.007G014200 | 6,356 | 4,646 | 4,353 | 40.5 | 12 | 387.2 | 155.5 | 1,450 | 161.876 | 11.5 | 7.351 | 0.216 |
| Gorai.007G035900 | 5,529 | 3,220 | 2,895 | 43.6 | 16 | 201.3 | 153.9 | 964 | 107.032 | 9 | 7.766 | 0.06 |
| Gorai.007G070500 | 6,029 | 2,743 | 2,058 | 41.3 | 18 | 152.4 | 193.3 | 685 | 75.029 | 26.5 | 9.691 | 0.141 |
| Gorai.007G108700 | 5,391 | 2,505 | 2,202 | 45.3 | 5 | 501 | 721.5 | 733 | 82.708 | 16 | 9.231 | -0.08 |
| Gorai.007G115900 | 7,664 | 3,919 | 3,759 | 40.1 | 22 | 178.1 | 178.3 | 1,252 | 142.373 | 16.5 | 8.265 | 0.091 |
| Gorai.007G116000 | 9,490 | 4,480 | 4,197 | 41.1 | 24 | 186.7 | 217.8 | 1,398 | 159.472 | 21 | 8.422 | -0.056 |
| Gorai.007G116700 | 5,355 | 4,030 | 3,888 | 43.5 | 12 | 335.8 | 120.5 | 1,295 | 139.481 | 1.5 | 6.757 | 0.104 |
| Gorai.007G128600 | 6,367 | 4,919 | 4,521 | 43 | 11 | 447.2 | 144.8 | 1,506 | 169.462 | 20.5 | 8.039 | 0.178 |
| Gorai.007G150300 | 8,551 | 5,420 | 4,632 | 42.7 | 13 | 416.9 | 257 | 1,543 | 171.079 | 15 | 7.815 | 0.23 |
| Gorai.007G230200 | 2,906 | 2,349 | 2,175 | 45 | 3 | 783 | 278.5 | 724 | 81.361 | 20 | 9.541 | -0.067 |
| Gorai.007G234000 | 5,286 | 2,379 | 2,073 | 42.3 | 8 | 297.4 | 415.3 | 690 | 76.782 | 5.5 | 7.379 | 0.02 |
| Gorai.007G236200 | 6,828 | 2,519 | 2,022 | 41.6 | 8 | 314.9 | 611.9 | 673 | 75.1 | 5 | 7.183 | 0.087 |
| Gorai.007G239200 | 18,268 | 5,148 | 4,008 | 42 | 27 | 190.7 | 504.6 | 1,335 | 150.087 | 38.5 | 9.495 | -0.077 |
| Gorai.007G244600 | 4,952 | 2,506 | 2,070 | 43.8 | 9 | 278.4 | 305.8 | 689 | 77.844 | -4.5 | 5.757 | -0.565 |
| Gorai.007G306900 | 5,020 | 3,998 | 3,804 | 43.2 | 12 | 333.2 | 92.9 | 1,267 | 140.394 | 12.5 | 7.626 | 0.184 |
| Gorai.007G310500 | 5,408 | 4,497 | 4,461 | 42.4 | 10 | 449.7 | 101.2 | 1,486 | 165.159 | -1 | 6.442 | 0.21 |
| Gorai.007G310600 | 5,702 | 4,712 | 4,410 | 42.2 | 11 | 428.4 | 99 | 1,469 | 163.082 | -5 | 6.128 | 0.233 |
| Gorai.007G310700 | 3,472 | 3,054 | 3,054 | 41.5 | 3 | 1,018.00 | 209 | 1,018 | 113.24 | 11 | 8.052 | 0.237 |
| Gorai.007G310800 | 5,811 | 4,670 | 4,359 | 42 | 9 | 518.9 | 142.6 | 1,452 | 161.22 | 7.5 | 7.116 | 0.258 |
| Gorai.007G374900 | 2,214 | 2,214 | 2,214 | 44.2 | 1 | 2,214.00 | No intron | 737 | 81.536 | 19 | 9.554 | 0.186 |
| Gorai.007G376200 | 2,362 | 1,270 | 990 | 47.5 | 7 | 181.4 | 182 | 329 | 36.697 | 9 | 9.148 | -0.269 |
| Gorai.008G047200 | 21,097 | 5,768 | 4,650 | 40.8 | 28 | 206 | 567.7 | 1,549 | 174.93 | 24.5 | 8.441 | 0.13 |
| Gorai.008G047300 | 22,240 | 4,886 | 4,548 | 41.5 | 27 | 181 | 667.5 | 1,515 | 170.601 | 20 | 8.209 | 0.096 |
| Gorai.008G047400 | 18,087 | 5,514 | 4,878 | 41.7 | 28 | 196.9 | 465.7 | 1,625 | 183.067 | 13.5 | 7.719 | 0.074 |
| Gorai.008G047500 | 14,016 | 5,479 | 4,872 | 42.5 | 27 | 202.9 | 328.3 | 1,623 | 182.398 | 3 | 6.794 | 0.067 |
| Gorai.008G058200 | 17,627 | 2,004 | 2,004 | 39 | 14 | 143.1 | 1,201.80 | 667 | 75.854 | 12.5 | 9.137 | 0.181 |
| Gorai.008G096100 | 21,367 | 5,035 | 4,020 | 42.2 | 26 | 193.7 | 652.6 | 1,339 | 150.192 | 45.5 | 9.684 | -0.072 |
| Gorai.008G134900 | 5,114 | 4,142 | 3,858 | 43.3 | 11 | 376.5 | 97.2 | 1,285 | 138.239 | 0.5 | 6.582 | 0.126 |
| Gorai.008G135100 | 6,136 | 4,347 | 3,831 | 43.4 | 12 | 362.3 | 103.3 | 1,276 | 137.386 | 6.5 | 7.027 | 0.179 |
| Gorai.008G145800 | 6,175 | 4,912 | 4,524 | 43 | 10 | 491.2 | 101.6 | 1,507 | 169.406 | 15 | 7.518 | 0.155 |
| Gorai.008G188000 | 3,381 | 2,488 | 2,073 | 47.2 | 5 | 497.6 | 223.3 | 690 | 76.425 | 19 | 9.082 | 0.047 |
| Gorai.008G191700 | 8,783 | 3,473 | 2,853 | 42.4 | 18 | 192.9 | 312.4 | 950 | 106.064 | 5.5 | 7.669 | -0.002 |
| Gorai.008G219100 | 5,624 | 3,998 | 3,570 | 41.1 | 9 | 444.2 | 203.3 | 1,189 | 133.058 | 13 | 7.292 | 0.086 |
| Gorai.008G219500 | 3,221 | 2,539 | 1,974 | 46.4 | 5 | 507.8 | 170.5 | 657 | 73.327 | 21 | 9.469 | 0.118 |
| Gorai.008G225400 | 6,517 | 4,217 | 3,747 | 43.9 | 12 | 351.4 | 209.1 | 1,248 | 136.674 | 23 | 9.125 | 0.16 |
| Gorai.008G271700 | 6,705 | 5,222 | 4,533 | 42.1 | 11 | 474.7 | 143.5 | 1,510 | 169.116 | 13 | 7.396 | 0.174 |
| Gorai.008G290900 | 8,241 | 2,160 | 2,160 | 43.4 | 8 | 270 | 868.7 | 719 | 81.538 | 23 | 9.479 | -0.066 |
| Gorai.009G022400 | 8,815 | 4,801 | 4,260 | 42.5 | 24 | 200 | 174.5 | 1,419 | 162.028 | 10.5 | 7.557 | 0.044 |
| Gorai.009G073800 | 5,424 | 2,522 | 2,061 | 44.2 | 9 | 280.2 | 362.8 | 686 | 76.412 | 18 | 9.279 | 0.114 |
| Gorai.009G110600 | 6,647 | 3,040 | 2,169 | 42.2 | 13 | 233.8 | 300.6 | 722 | 79.653 | 16 | 8.682 | 0.259 |
| Gorai.009G120300 | 8,108 | 4,908 | 4,359 | 43.5 | 19 | 258.3 | 177.8 | 1,452 | 164.883 | 15.5 | 8.062 | 0.049 |
| Gorai.009G123500 | 22,674 | 4,871 | 4,389 | 40.1 | 36 | 135.3 | 508.7 | 1,462 | 162.805 | 10 | 7.082 | 0.277 |
| Gorai.009G128700 | 7,130 | 4,463 | 4,314 | 42.5 | 24 | 186 | 116 | 1,437 | 163.696 | 12 | 7.672 | 0.031 |
| Gorai.009G128800 | 7,830 | 4,956 | 4,404 | 41.8 | 24 | 206.5 | 125 | 1,467 | 165.849 | 12.5 | 7.903 | 0.055 |
| Gorai.009G129000 | 7,743 | 5,068 | 4,410 | 42.1 | 24 | 211.2 | 116.3 | 1,469 | 165.483 | 10.5 | 7.589 | 0.071 |
| Gorai.009G137200 | 9,223 | 5,215 | 4,476 | 43.8 | 22 | 237 | 190.9 | 1,491 | 168.947 | 20 | 8.604 | 0.003 |
| Gorai.009G290300 | 5,566 | 2,823 | 2,007 | 46.6 | 3 | 941 | 1,371.50 | 668 | 73.584 | 27.5 | 9.785 | 0.115 |
| Gorai.009G302000 | 5,640 | 1,615 | 981 | 46.3 | 8 | 201.9 | 575 | 326 | 35.478 | 0 | 6.521 | -0.06 |
| Gorai.009G304900 | 8,091 | 4,687 | 4,470 | 42.9 | 18 | 260.4 | 200.2 | 1,489 | 168.328 | 16 | 7.993 | 0.04 |
| Gorai.009G342400 | 2,542 | 1,728 | 1,053 | 40.8 | 5 | 345.6 | 203.5 | 350 | 38.358 | -5.5 | 5.158 | 0.077 |
| Gorai.009G401200 | 4,262 | 2,148 | 1,929 | 42.8 | 10 | 214.8 | 234.9 | 642 | 71.018 | 18.5 | 9.45 | 0.256 |
| Gorai.009G433300 | 3,342 | 2,364 | 1,890 | 41.6 | 4 | 591 | 326 | 629 | 70.045 | 10 | 8.698 | 0.143 |
| Gorai.010G002700 | 5,096 | 4,207 | 3,729 | 43.7 | 10 | 420.7 | 98.8 | 1,242 | 134.901 | 9.5 | 7.384 | 0.157 |
| Gorai.010G067900 | 8,734 | 4,818 | 4,263 | 43 | 24 | 200.8 | 170.3 | 1,420 | 162.054 | 16.5 | 8.215 | 0.046 |
| Gorai.010G076800 | 6,264 | 4,195 | 3,735 | 44.6 | 9 | 466.1 | 258.6 | 1,244 | 136.473 | 24.5 | 9.338 | 0.077 |
| Gorai.010G111900 | 1,569 | 1,267 | 528 | 45.5 | 2 | 633.5 | 302 | 175 | 19.927 | 5.5 | 9.904 | -0.046 |
| Gorai.010G181700 | 3,837 | 1,920 | 1,815 | 43.1 | 7 | 274.3 | 186.3 | 604 | 67.689 | 9 | 8.54 | -0.221 |
| Gorai.011G034500 | 5,831 | 4,483 | 3,888 | 44.3 | 11 | 407.5 | 134.8 | 1,295 | 139.768 | 4.5 | 6.986 | 0.129 |
| Gorai.011G037200 | 1,568 | 1,285 | 690 | 47.2 | 2 | 642.5 | 283 | 229 | 25.772 | 13.5 | 10.741 | -0.104 |
| Gorai.011G057300 | 6,217 | 5,025 | 4,845 | 43.7 | 9 | 558.3 | 149 | 1,614 | 177.726 | 24.5 | 7.702 | -0.125 |
| Gorai.011G066600 | 5,857 | 4,488 | 4,254 | 40.9 | 13 | 345.2 | 114.1 | 1,417 | 158.595 | 10 | 7.777 | 0.254 |
| Gorai.011G066700 | 5,744 | 4,660 | 3,876 | 41 | 9 | 517.8 | 135.5 | 1,292 | 144.336 | 11.5 | 8.026 | 0.328 |
| Gorai.011G066800 | 5,749 | 3,823 | 3,594 | 42 | 12 | 318.6 | 175.1 | 1,197 | 132.825 | 5 | 7.309 | 0.214 |
| Gorai.011G066900 | 4,908 | 3,393 | 2,868 | 40.3 | 7 | 484.7 | 252.5 | 955 | 105.93 | -10.5 | 5.24 | 0.293 |
| Gorai.011G071500 | 8,307 | 4,825 | 4,341 | 41.9 | 20 | 241.3 | 183.3 | 1,446 | 163.456 | 29 | 9.032 | 0.024 |
| Gorai.011G071700 | 8,482 | 5,134 | 4,467 | 43.3 | 21 | 244.5 | 167.4 | 1,488 | 168.213 | 6.5 | 7.131 | 0.017 |
| Gorai.011G079200 | 6,858 | 4,484 | 4,299 | 41.6 | 24 | 186.8 | 103.2 | 1,432 | 161.757 | 10.5 | 7.74 | 0.087 |
| Gorai.011G086500 | 3,771 | 1,637 | 828 | 41.7 | 11 | 148.8 | 213.4 | 275 | 30.639 | 4 | 8.23 | -0.08 |
| Gorai.011G166900 | 3,479 | 840 | 600 | 45.5 | 5 | 168 | 659.8 | 199 | 21.552 | -3 | 4.8 | -0.117 |
| Gorai.011G204900 | 2,866 | 1,824 | 1,824 | 41 | 9 | 202.7 | 130.3 | 607 | 68.677 | 2 | 6.936 | 0.223 |
| Gorai.011G205000 | 6,549 | 2,802 | 2,802 | 40.9 | 17 | 164.8 | 234.2 | 933 | 105.563 | 4 | 6.935 | -0.103 |
| Gorai.011G205700 | 3,605 | 2,855 | 2,715 | 45.6 | 6 | 475.8 | 150 | 904 | 98.793 | 44.5 | 10.368 | 0.068 |
| Gorai.011G220300 | 3,354 | 1,280 | 780 | 45.5 | 3 | 426.7 | 1,037.00 | 259 | 28.305 | 2.5 | 7.731 | 0.004 |
| Gorai.011G238200 | 6,203 | 4,204 | 3,324 | 42.6 | 14 | 300.3 | 153.8 | 1,107 | 123.173 | 40.5 | 8.834 | -0.148 |
| Gorai.011G252900 | 8,131 | 5,096 | 4,221 | 43.7 | 11 | 463.3 | 303.5 | 1,406 | 155.853 | 1 | 6.566 | 0.071 |
| Gorai.011G295700 | 6,238 | 5,063 | 4,539 | 40.7 | 12 | 421.9 | 106.8 | 1,512 | 168.96 | 15 | 7.765 | 0.133 |
| Gorai.012G023600 | 7,065 | 4,633 | 4,464 | 42.3 | 20 | 231.7 | 128 | 1,487 | 168.404 | 7.5 | 7.06 | 0.039 |
| Gorai.012G034800 | 2,349 | 402 | 402 | 44.3 | 5 | 80.4 | 486.8 | 133 | 14.476 | -2.5 | 4.849 | 0.096 |
| Gorai.012G035000 | 3,975 | 1,184 | 789 | 42.7 | 6 | 197.3 | 558.2 | 262 | 28.612 | 5 | 8.803 | -0.056 |
| Gorai.012G036300 | 2,302 | 1,993 | 1,620 | 42.7 | 2 | 996.5 | 309 | 539 | 60.419 | 16 | 9.227 | 0.132 |
| Gorai.012G038700 | 6,829 | 2,317 | 1,935 | 42.8 | 18 | 128.7 | 265.4 | 644 | 69.608 | 4 | 7.648 | 0.089 |
| Gorai.012G058100 | 6,957 | 4,566 | 3,750 | 42.7 | 8 | 570.8 | 341.6 | 1,249 | 136.463 | 16.5 | 8.164 | 0.133 |
| Gorai.012G067200 | 2,247 | 2,247 | 2,247 | 43.7 | 1 | 2,247.00 | No intron | 748 | 82.546 | 14 | 9.386 | 0.171 |
| Gorai.012G067300 | 2,331 | 2,331 | 2,232 | 43.7 | 1 | 2,331.00 | No intron | 743 | 82.37 | 15.5 | 9.463 | 0.129 |
| Gorai.012G069000 | 8,699 | 5,161 | 4,224 | 43.1 | 11 | 469.2 | 320.5 | 1,407 | 156.145 | -5.5 | 6.248 | 0.069 |
| Gorai.012G091700 | 2,253 | 2,253 | 2,253 | 43.9 | 1 | 2,253.00 | No intron | 750 | 82.947 | 16 | 9.611 | 0.156 |
| Gorai.012G097300 | 13,940 | 4,419 | 4,155 | 41.2 | 24 | 184.1 | 414 | 1,384 | 156.092 | 14.5 | 7.909 | 0.106 |
| Gorai.012G174600 | 8,628 | 4,830 | 4,365 | 41.6 | 10 | 483 | 422 | 1,454 | 163.597 | 10 | 7.356 | 0.137 |
| Gorai.013G023900 | 2,031 | 2,031 | 2,031 | 42.1 | 1 | 2,031.00 | No intron | 676 | 75.351 | 16.5 | 9.361 | 0.261 |
| Gorai.013G066600 | 4,596 | 3,291 | 3,291 | 39.1 | 18 | 182.8 | 76.8 | 1,096 | 122.344 | 38.5 | 9.545 | 0.112 |
| Gorai.013G110000 | 7,155 | 4,614 | 4,338 | 41.8 | 24 | 192.3 | 110.5 | 1,445 | 164.054 | 17.5 | 8.417 | 0.041 |
| Gorai.013G154700 | 5,839 | 4,911 | 4,491 | 41.8 | 10 | 491.1 | 103.1 | 1,496 | 165.594 | 7 | 7.032 | 0.299 |
| Gorai.013G154800 | 5,840 | 4,823 | 4,446 | 41 | 10 | 482.3 | 113 | 1,481 | 165.145 | 3.5 | 6.836 | 0.223 |
| Gorai.013G163700 | 4,467 | 3,848 | 3,759 | 42.5 | 7 | 549.7 | 103.2 | 1,252 | 137.604 | 31.5 | 9.615 | 0.037 |
| Gorai.013G209700 | 3,878 | 1,845 | 1,845 | 38.5 | 14 | 131.8 | 156.4 | 614 | 69.722 | 7 | 7.794 | 0.207 |
| Gorai.013G267600 | 7,819 | 3,613 | 3,249 | 41.7 | 15 | 240.9 | 300.4 | 1,082 | 119.395 | 34.5 | 8.839 | -0.151 |
| Gorai.N013800 | 6,807 | 4,259 | 4,110 | 42.5 | 23 | 185.2 | 115.8 | 1,369 | 153.965 | 4 | 6.824 | 0.169 |
