# Supplementary material for: RNA-Sequencing, Physiological and RNAi Analyses Provide Insights into the Response Mechanism of the ABC-Mediated Resistance to Verticillium dahliae Infection in Cotton
Source: Genes (Basel). 2019 Feb 1;10(2):110. doi: 10.3390/genes10020110 (PMC6410047; doi:10.3390/genes10020110)
Supplement: Supplementary file 1 [file genes-10-00110-s001.zip › Supplementary files/Supplementary Table 3. Subcellular localization prediction of ABC proteins in Gossypium raimondii..docx]

**Supplementary Table 3.** Subcellular localization prediction of ABC proteins in *Gossypium raimondii.*

| Name | Chromosome Locations | | | TargetP | | | | | Pprowler | WoLF PSORT |
| --- | --- | --- | --- | --- | --- | --- | --- | --- | --- | --- |
|  | Chr. | Start | End | cTP | mTP | SP | other | Loc |  |  |
| Gorai.001G003100 | Chr01 | 264,426 | 271,839 | 0.112 | 0.204 | 0.042 | 0.861 | _ | OTHER | plas |
| Gorai.001G057200 | Chr01 | 5,592,430 | 5,599,949 | 0.201 | 0.399 | 0.054 | 0.318 | M | MTP | plas |
| Gorai.001G057400 | Chr01 | 5,646,760 | 5,654,308 | 0.239 | 0.218 | 0.088 | 0.506 | _ | OTHER | plas |
| Gorai.001G068300 | Chr01 | 6,856,295 | 6,862,236 | 0.434 | 0.044 | 0.093 | 0.694 | _ | OTHER | plas |
| Gorai.001G075900 | Chr01 | 7,809,608 | 7,814,554 | 0.059 | 0.526 | 0.006 | 0.553 | _ | OTHER | plas |
| Gorai.001G115700 | Chr01 | 13,703,544 | 13,737,700 | 0.193 | 0.071 | 0.044 | 0.887 | _ | OTHER | plas |
| Gorai.001G116000 | Chr01 | 13,772,731 | 13,783,951 | 0.359 | 0.047 | 0.031 | 0.852 | _ | OTHER | plas |
| Gorai.001G133500 | Chr01 | 17,131,792 | 17,136,040 | 0.199 | 0.089 | 0.07 | 0.872 | _ | OTHER | cyto |
| Gorai.001G147100 | Chr01 | 20,001,174 | 20,012,216 | 0.068 | 0.2 | 0.069 | 0.917 | _ | OTHER | plas |
| Gorai.001G147200 | Chr01 | 20,134,737 | 20,139,203 | 0.148 | 0.186 | 0.032 | 0.89 | _ | OTHER | cyto |
| Gorai.001G182400 | Chr01 | 27,898,390 | 27,907,981 | 0.838 | 0.023 | 0.076 | 0.2 | C | CTP | plas |
| Gorai.001G249000 | Chr01 | 50,540,064 | 50,542,322 | 0.222 | 0.047 | 0.042 | 0.816 | _ | OTHER | plas |
| Gorai.001G254500 | Chr01 | 52,612,325 | 52,614,784 | 0.064 | 0.157 | 0.06 | 0.937 | _ | OTHER | nucl |
| Gorai.001G256100 | Chr01 | 52,856,974 | 52,863,511 | 0.06 | 0.066 | 0.222 | 0.959 | _ | SP | plas |
| Gorai.001G258400 | Chr01 | 53,274,097 | 53,279,975 | 0.012 | 0.128 | 0.186 | 0.943 | _ | OTHER | plas |
| Gorai.001G258500 | Chr01 | 53,282,544 | 53,287,141 | 0.039 | 0.114 | 0.116 | 0.963 | _ | OTHER | plas |
| Gorai.002G023500 | Chr02 | 1,672,961 | 1,679,792 | 0.432 | 0.072 | 0.017 | 0.102 | C | OTHER | chlo |
| Gorai.002G057800 | Chr02 | 6,200,507 | 6,212,938 | 0.752 | 0.118 | 0.053 | 0.142 | C | CTP | plas |
| Gorai.002G062500 | Chr02 | 7,387,128 | 7,389,098 | 0.038 | 0.353 | 0.03 | 0.837 | _ | OTHER | plas |
| Gorai.002G073600 | Chr02 | 8,603,180 | 8,605,749 | 0.056 | 0.112 | 0.061 | 0.939 | _ | OTHER | plas |
| Gorai.002G146400 | Chr02 | 26,941,850 | 26,964,903 | 0.005 | 0.79 | 0.046 | 0.13 | M | MTP | plas |
| Gorai.002G153900 | Chr02 | 31,528,374 | 31,535,314 | 0.652 | 0.045 | 0.003 | 0.462 | C | CTP | plas |
| Gorai.002G162300 | Chr02 | 37,674,805 | 37,681,093 | 0.017 | 0.171 | 0.886 | 0.011 | S | SP | plas |
| Gorai.002G180700 | Chr02 | 47,537,930 | 47,545,936 | 0.012 | 0.197 | 0.364 | 0.364 | S | OTHER | plas |
| Gorai.002G186500 | Chr02 | 49,741,429 | 49,749,904 | 0.276 | 0.091 | 0.013 | 0.046 | C | MTP | plas |
| Gorai.002G188900 | Chr02 | 50,881,514 | 50,885,882 | 0.011 | 0.106 | 0.098 | 0.874 | _ | OTHER | plas |
| Gorai.002G233500 | Chr02 | 59,325,349 | 59,331,229 | 0.545 | 0.035 | 0.009 | 0.419 | C | CTP | plas |
| Gorai.002G246800 | Chr02 | 61,077,195 | 61,083,223 | 0.137 | 0.104 | 0.102 | 0.824 | _ | OTHER | plas |
| Gorai.002G252600 | Chr02 | 61,568,386 | 61,570,517 | 0.063 | 0.154 | 0.016 | 0.82 | _ | OTHER | plas |
| Gorai.003G038900 | Chr03 | 4,183,511 | 4,194,201 | 0.041 | 0.831 | 0.029 | 0.118 | M | MTP | plas |
| Gorai.003G047600 | Chr03 | 6,476,573 | 6,481,197 | 0.757 | 0.528 | 0.002 | 0.038 | C | MTP | cyto |
| Gorai.003G062100 | Chr03 | 11,312,099 | 11,321,009 | 0.272 | 0.264 | 0.044 | 0.512 | _ | OTHER | plas |
| Gorai.003G064800 | Chr03 | 12,782,431 | 12,795,469 | 0.272 | 0.244 | 0.163 | 0.339 | _ | OTHER | plas |
| Gorai.003G070300 | Chr03 | 15,764,114 | 15,766,419 | 0.631 | 0.054 | 0.007 | 0.519 | C | CTP | plas |
| Gorai.003G082700 | Chr03 | 20,353,357 | 20,360,683 | 0.078 | 0.076 | 0.013 | 0.879 | _ | SP | plas |
| Gorai.003G089800 | Chr03 | 26,054,546 | 26,058,472 | 0.126 | 0.087 | 0.085 | 0.917 | _ | OTHER | plas |
| Gorai.003G144400 | Chr03 | 41,054,249 | 41,061,055 | 0.778 | 0.086 | 0.013 | 0.23 | C | CTP | plas |
| Gorai.003G153100 | Chr03 | 42,145,515 | 42,156,854 | 0.031 | 0.926 | 0.003 | 0.036 | M | MTP | chlo |
| Gorai.003G183800 | Chr03 | 45,492,558 | 45,500,859 | 0.011 | 0.03 | 0.84 | 0.349 | S | SP | plas |
| Gorai.004G033800 | Chr04 | 2,770,386 | 2,773,465 | 0.14 | 0.123 | 0.135 | 0.882 | _ | OTHER | plas |
| Gorai.004G089200 | Chr04 | 12,007,730 | 12,013,346 | 0.303 | 0.006 | 0.146 | 0.646 | _ | SP | plas |
| Gorai.004G111000 | Chr04 | 23,881,223 | 23,888,372 | 0.525 | 0.08 | 0.073 | 0.565 | _ | OTHER | plas |
| Gorai.004G111900 | Chr04 | 25,424,671 | 25,430,699 | 0.939 | 0.037 | 0.004 | 0.044 | C | CTP | chlo |
| Gorai.004G131200 | Chr04 | 35,549,122 | 35,557,311 | 0.026 | 0.154 | 0.086 | 0.911 | _ | OTHER | cyto |
| Gorai.004G138100 | Chr04 | 38,645,602 | 38,656,531 | 0.114 | 0.551 | 0.029 | 0.371 | M | MTP | plas |
| Gorai.004G147200 | Chr04 | 41,464,303 | 41,470,397 | 0.097 | 0.181 | 0.056 | 0.881 | _ | OTHER | plas |
| Gorai.004G177600 | Chr04 | 48,568,469 | 48,573,730 | 0.136 | 0.077 | 0.069 | 0.928 | _ | OTHER | cyto |
| Gorai.004G178100 | Chr04 | 48,631,249 | 48,634,921 | 0.687 | 0.127 | 0.018 | 0.495 | C | OTHER | plas |
| Gorai.004G261400 | Chr04 | 59,676,283 | 59,681,790 | 0.76 | 0.047 | 0.123 | 0.254 | C | CTP | chlo |
| Gorai.005G134100 | Chr05 | 33,859,046 | 33,862,807 | 0.431 | 0.079 | 0.094 | 0.643 | _ | OTHER | plas |
| Gorai.005G172300 | Chr05 | 50,097,493 | 50,103,851 | 0.004 | 0.155 | 0.673 | 0.345 | S | SP | plas |
| Gorai.005G214200 | Chr05 | 59,679,447 | 59,684,136 | 0.068 | 0.085 | 0.11 | 0.941 | _ | OTHER | plas |
| Gorai.005G221600 | Chr05 | 60,424,839 | 60,427,434 | 0.206 | 0.047 | 0.078 | 0.919 | _ | OTHER | plas |
| Gorai.005G221800 | Chr05 | 60,437,644 | 60,438,929 | 0.672 | 0.036 | 0.05 | 0.649 | C | OTHER | mito |
| Gorai.005G221900 | Chr05 | 60,453,996 | 60,456,300 | 0.132 | 0.095 | 0.152 | 0.772 | _ | OTHER | plas |
| Gorai.005G222100 | Chr05 | 60,489,413 | 60,492,272 | 0.322 | 0.257 | 0.049 | 0.345 | _ | MTP | plas |
| Gorai.005G222400 | Chr05 | 60,520,371 | 60,523,437 | 0.174 | 0.112 | 0.076 | 0.712 | _ | OTHER | plas |
| Gorai.006G001900 | Chr06 | 532,935 | 534,970 | 0.031 | 0.133 | 0.036 | 0.926 | _ | OTHER | plas |
| Gorai.006G021600 | Chr06 | 5,497,978 | 5,504,204 | 0.052 | 0.076 | 0.218 | 0.963 | _ | SP | plas |
| Gorai.006G026800 | Chr06 | 6,899,849 | 6,904,242 | 0.035 | 0.103 | 0.216 | 0.921 | _ | OTHER | plas |
| Gorai.006G033600 | Chr06 | 8,835,983 | 8,841,278 | 0.021 | 0.855 | 0.001 | 0.386 | M | SP | plas |
| Gorai.006G124200 | Chr06 | 37,586,845 | 37,589,159 | 0.831 | 0.019 | 0.004 | 0.373 | C | CTP | plas |
| Gorai.006G124600 | Chr06 | 37,640,051 | 37,643,738 | 0.248 | 0.098 | 0.071 | 0.84 | _ | OTHER | cyto |
| Gorai.006G126900 | Chr06 | 37,942,333 | 37,949,109 | 0.161 | 0.1 | 0.022 | 0.93 | _ | OTHER | plas |
| Gorai.006G130900 | Chr06 | 38,488,064 | 38,495,022 | 0.078 | 0.283 | 0.016 | 0.72 | _ | OTHER | plas |
| Gorai.006G147000 | Chr06 | 40,530,746 | 40,536,980 | 0.234 | 0.055 | 0.005 | 0.736 | _ | OTHER | plas |
| Gorai.006G157100 | Chr06 | 41,606,396 | 41,612,778 | 0.357 | 0.385 | 0.047 | 0.271 | M | MTP | plas |
| Gorai.006G157200 | Chr06 | 41,622,746 | 41,625,716 | 0.357 | 0.385 | 0.047 | 0.271 | M | MTP | nucl |
| Gorai.006G163000 | Chr06 | 42,321,770 | 42,328,309 | 0.354 | 0.018 | 0.237 | 0.89 | _ | SP | plas |
| Gorai.007G014200 | Chr07 | 1,110,506 | 1,116,861 | 0.134 | 0.022 | 0.037 | 0.955 | _ | SP | plas |
| Gorai.007G035900 | Chr07 | 2,456,193 | 2,461,721 | 0.008 | 0.752 | 0.104 | 0.101 | M | SP | plas |
| Gorai.007G070500 | Chr07 | 4,973,122 | 4,979,150 | 0.015 | 0.303 | 0.444 | 0.098 | S | SP | plas |
| Gorai.007G108700 | Chr07 | 8,203,958 | 8,209,348 | 0.102 | 0.292 | 0.041 | 0.593 | _ | OTHER | plas |
| Gorai.007G115900 | Chr07 | 9,011,724 | 9,019,387 | 0.058 | 0.628 | 0.02 | 0.381 | M | MTP | plas |
| Gorai.007G116000 | Chr07 | 9,026,964 | 9,036,453 | 0.39 | 0.069 | 0.078 | 0.748 | _ | OTHER | plas |
| Gorai.007G116700 | Chr07 | 9,129,421 | 9,134,775 | 0.083 | 0.153 | 0.057 | 0.901 | _ | OTHER | plas |
| Gorai.007G128600 | Chr07 | 10,270,056 | 10,276,422 | 0.329 | 0.019 | 0.126 | 0.536 | _ | SP | plas |
| Gorai.007G150300 | Chr07 | 12,718,571 | 12,727,121 | 0.604 | 0.024 | 0.111 | 0.168 | C | SP | plas |
| Gorai.007G230200 | Chr07 | 28,861,792 | 28,864,697 | 0.069 | 0.127 | 0.065 | 0.916 | _ | OTHER | plas |
| Gorai.007G234000 | Chr07 | 30,334,429 | 30,339,714 | 0.489 | 0.059 | 0.04 | 0.731 | _ | OTHER | plas |
| Gorai.007G236200 | Chr07 | 31,730,409 | 31,737,236 | 0.129 | 0.079 | 0.078 | 0.924 | _ | OTHER | plas |
| Gorai.007G239200 | Chr07 | 32,698,794 | 32,717,061 | 0.015 | 0.951 | 0.007 | 0.134 | M | MTP | plas |
| Gorai.007G244600 | Chr07 | 36,467,661 | 36,472,612 | 0.599 | 0.678 | 0.002 | 0.026 | M | MTP | chlo |
| Gorai.007G306900 | Chr07 | 52,043,607 | 52,048,626 | 0.284 | 0.059 | 0.041 | 0.869 | _ | OTHER | plas |
| Gorai.007G310500 | Chr07 | 52,422,123 | 52,427,530 | 0.01 | 0.056 | 0.825 | 0.048 | S | SP | plas |
| Gorai.007G310600 | Chr07 | 52,442,909 | 52,448,610 | 0.163 | 0.099 | 0.13 | 0.351 | _ | SP | plas |
| Gorai.007G310700 | Chr07 | 52,465,675 | 52,469,146 | 0.069 | 0.066 | 0.353 | 0.276 | S | SP | plas |
| Gorai.007G310800 | Chr07 | 52,491,129 | 52,496,939 | 0.08 | 0.061 | 0.291 | 0.417 | _ | SP | plas |
| Gorai.007G374900 | Chr07 | 60,672,472 | 60,674,685 | 0.213 | 0.1 | 0.022 | 0.83 | _ | OTHER | plas |
| Gorai.007G376200 | Chr07 | 60,744,887 | 60,747,248 | 0.188 | 0.08 | 0.089 | 0.789 | _ | OTHER | cyto |
| Gorai.008G047200 | Chr08 | 6,388,142 | 6,409,238 | 0.002 | 0.124 | 0.102 | 0.838 | _ | SP | plas |
| Gorai.008G047300 | Chr08 | 6,448,515 | 6,470,754 | 0.004 | 0.187 | 0.067 | 0.962 | _ | OTHER | plas |
| Gorai.008G047400 | Chr08 | 6,488,416 | 6,506,502 | 0.003 | 0.196 | 0.122 | 0.855 | _ | SP | plas |
| Gorai.008G047500 | Chr08 | 6,522,335 | 6,536,350 | 0.02 | 0.186 | 0.049 | 0.77 | _ | SP | plas |
| Gorai.008G058200 | Chr08 | 9,086,309 | 9,103,935 | 0.033 | 0.238 | 0.023 | 0.566 | _ | SP | plas |
| Gorai.008G096100 | Chr08 | 26,270,019 | 26,291,385 | 0.031 | 0.678 | 0.05 | 0.21 | M | MTP | plas |
| Gorai.008G134900 | Chr08 | 38,276,163 | 38,281,276 | 0.197 | 0.049 | 0.052 | 0.912 | _ | OTHER | plas |
| Gorai.008G135100 | Chr08 | 38,301,864 | 38,307,999 | 0.061 | 0.03 | 0.157 | 0.963 | _ | OTHER | plas |
| Gorai.008G145800 | Chr08 | 39,833,751 | 39,839,925 | 0.479 | 0.025 | 0.259 | 0.47 | C | SP | plas |
| Gorai.008G188000 | Chr08 | 46,962,622 | 46,966,002 | 0.844 | 0.021 | 0.006 | 0.271 | C | CTP | plas |
| Gorai.008G191700 | Chr08 | 47,460,489 | 47,469,271 | 0.144 | 0.402 | 0.016 | 0.388 | M | MTP | plas |
| Gorai.008G219100 | Chr08 | 50,551,761 | 50,557,384 | 0.181 | 0.74 | 0.005 | 0.035 | M | MTP | plas |
| Gorai.008G219500 | Chr08 | 50,588,959 | 50,592,179 | 0.477 | 0.071 | 0.026 | 0.686 | _ | OTHER | plas |
| Gorai.008G225400 | Chr08 | 51,190,466 | 51,196,982 | 0.02 | 0.034 | 0.094 | 0.995 | _ | SP | plas |
| Gorai.008G271700 | Chr08 | 55,088,443 | 55,095,147 | 0.093 | 0.029 | 0.034 | 0.983 | _ | SP | plas |
| Gorai.008G290900 | Chr08 | 56,536,152 | 56,544,392 | 0.095 | 0.066 | 0.09 | 0.927 | _ | OTHER | plas |
| Gorai.009G022400 | Chr09 | 1,709,124 | 1,717,938 | 0.465 | 0.091 | 0.034 | 0.671 | _ | OTHER | plas |
| Gorai.009G073800 | Chr09 | 5,286,572 | 5,291,995 | 0.142 | 0.06 | 0.044 | 0.899 | _ | OTHER | plas |
| Gorai.009G110600 | Chr09 | 8,071,170 | 8,077,816 | 0.571 | 0.105 | 0.023 | 0.361 | C | CTP | plas |
| Gorai.009G120300 | Chr09 | 8,897,129 | 8,905,236 | 0.36 | 0.346 | 0.063 | 0.287 | C | OTHER | plas |
| Gorai.009G123500 | Chr09 | 9,215,346 | 9,238,019 | 0.008 | 0.105 | 0.297 | 0.613 | _ | SP | plas |
| Gorai.009G128700 | Chr09 | 9,672,777 | 9,679,906 | 0.076 | 0.608 | 0.032 | 0.413 | M | MTP | plas |
| Gorai.009G128800 | Chr09 | 9,682,477 | 9,690,306 | 0.652 | 0.278 | 0.023 | 0.083 | C | MTP | plas |
| Gorai.009G129000 | Chr09 | 9,702,635 | 9,710,377 | 0.525 | 0.268 | 0.043 | 0.166 | C | MTP | plas |
| Gorai.009G137200 | Chr09 | 10,339,243 | 10,348,465 | 0.65 | 0.484 | 0.014 | 0.082 | C | MTP | plas |
| Gorai.009G290300 | Chr09 | 24,986,745 | 24,992,310 | 0.157 | 0.087 | 0.037 | 0.892 | _ | MTP | plas |
| Gorai.009G302000 | Chr09 | 27,064,346 | 27,069,985 | 0.948 | 0.099 | 0.005 | 0.02 | C | CTP | chlo |
| Gorai.009G304900 | Chr09 | 27,600,406 | 27,608,496 | 0.524 | 0.4 | 0.003 | 0.105 | C | MTP | plas |
| Gorai.009G342400 | Chr09 | 39,970,027 | 39,972,568 | 0.014 | 0.201 | 0.488 | 0.747 | _ | OTHER | chlo |
| Gorai.009G401200 | Chr09 | 57,876,189 | 57,880,450 | 0.243 | 0.71 | 0.001 | 0.081 | M | MTP | plas |
| Gorai.009G433300 | Chr09 | 68,074,930 | 68,078,271 | 0.038 | 0.179 | 0.068 | 0.932 | _ | OTHER | plas |
| Gorai.010G002700 | Chr10 | 108,468 | 113,563 | 0.121 | 0.019 | 0.124 | 0.964 | _ | OTHER | plas |
| Gorai.010G067900 | Chr10 | 8,839,937 | 8,848,670 | 0.34 | 0.103 | 0.065 | 0.712 | _ | OTHER | plas |
| Gorai.010G076800 | Chr10 | 11,063,764 | 11,070,027 | 0.035 | 0.157 | 0.092 | 0.946 | _ | SP | plas |
| Gorai.010G111900 | Chr10 | 21,263,837 | 21,265,405 | 0.003 | 0.633 | 0.172 | 0.815 | _ | MTP | cyto |
| Gorai.010G181700 | Chr10 | 52,950,537 | 52,954,373 | 0.01 | 0.711 | 0.004 | 0.189 | M | MTP | chlo |
| Gorai.011G034500 | Chr11 | 2,548,209 | 2,554,039 | 0.063 | 0.062 | 0.054 | 0.965 | _ | OTHER | plas |
| Gorai.011G037200 | Chr11 | 2,796,470 | 2,798,037 | 0.217 | 0.31 | 0.056 | 0.174 | M | MTP | mito |
| Gorai.011G057300 | Chr11 | 4,573,905 | 4,580,121 | 0.593 | 0.242 | 0.007 | 0.444 | C | CTP | plas |
| Gorai.011G066600 | Chr11 | 5,684,750 | 5,690,606 | 0.002 | 0.137 | 0.549 | 0.57 | _ | SP | plas |
| Gorai.011G066700 | Chr11 | 5,743,171 | 5,748,914 | 0.002 | 0.107 | 0.065 | 0.947 | _ | SP | plas |
| Gorai.011G066800 | Chr11 | 5,790,323 | 5,796,071 | 0.002 | 0.048 | 0.574 | 0.99 | _ | SP | plas |
| Gorai.011G066900 | Chr11 | 5,818,161 | 5,823,068 | 0.003 | 0.065 | 0.878 | 0.309 | S | SP | plas |
| Gorai.011G071500 | Chr11 | 6,599,477 | 6,607,783 | 0.764 | 0.119 | 0.054 | 0.227 | C | OTHER | plas |
| Gorai.011G071700 | Chr11 | 6,657,101 | 6,665,582 | 0.603 | 0.587 | 0.011 | 0.062 | C | MTP | plas |
| Gorai.011G079200 | Chr11 | 7,895,042 | 7,901,899 | 0.292 | 0.553 | 0.016 | 0.207 | M | MTP | plas |
| Gorai.011G086500 | Chr11 | 9,032,069 | 9,035,839 | 0.634 | 0.071 | 0.116 | 0.174 | C | CTP | chlo |
| Gorai.011G166900 | Chr11 | 33,318,553 | 33,322,031 | 0.106 | 0.05 | 0.156 | 0.756 | _ | SP | cyto |
| Gorai.011G204900 | Chr11 | 49,631,005 | 49,633,870 | 0.031 | 0.394 | 0.128 | 0.651 | _ | OTHER | plas |
| Gorai.011G205000 | Chr11 | 49,636,145 | 49,642,693 | 0.196 | 0.12 | 0.154 | 0.865 | _ | OTHER | plas |
| Gorai.011G205700 | Chr11 | 49,721,311 | 49,724,915 | 0.162 | 0.163 | 0.025 | 0.866 | _ | OTHER | plas |
| Gorai.011G220300 | Chr11 | 52,858,172 | 52,861,525 | 0.303 | 0.037 | 0.075 | 0.908 | _ | OTHER | golg |
| Gorai.011G238200 | Chr11 | 56,066,008 | 56,072,210 | 0.002 | 0.088 | 0.933 | 0.056 | S | SP | plas |
| Gorai.011G252900 | Chr11 | 58,147,796 | 58,155,926 | 0.172 | 0.297 | 0.237 | 0.463 | _ | SP | plas |
| Gorai.011G295700 | Chr11 | 62,607,890 | 62,614,127 | 0.007 | 0.037 | 0.588 | 0.734 | _ | SP | plas |
| Gorai.012G023600 | Chr12 | 2,913,759 | 2,920,823 | 0.935 | 0.232 | 0.029 | 0.034 | C | CTP | plas |
| Gorai.012G034800 | Chr12 | 4,294,383 | 4,296,731 | 0.459 | 0.556 | 0.039 | 0.031 | M | MTP | chlo |
| Gorai.012G035000 | Chr12 | 4,328,673 | 4,332,647 | 0.443 | 0.6 | 0.012 | 0.042 | M | MTP | chlo |
| Gorai.012G036300 | Chr12 | 4,493,532 | 4,495,833 | 0.092 | 0.13 | 0.032 | 0.88 | _ | OTHER | plas |
| Gorai.012G038700 | Chr12 | 4,816,893 | 4,823,721 | 0.326 | 0.052 | 0.028 | 0.81 | _ | OTHER | plas |
| Gorai.012G058100 | Chr12 | 8,049,879 | 8,056,835 | 0.014 | 0.31 | 0.122 | 0.884 | _ | SP | plas |
| Gorai.012G067200 | Chr12 | 9,646,865 | 9,649,111 | 0.56 | 0.032 | 0.063 | 0.72 | _ | OTHER | plas |
| Gorai.012G067300 | Chr12 | 9,663,896 | 9,666,226 | 0.226 | 0.134 | 0.047 | 0.728 | _ | OTHER | plas |
| Gorai.012G069000 | Chr12 | 10,116,306 | 10,125,004 | 0.166 | 0.348 | 0.152 | 0.419 | _ | MTP | plas |
| Gorai.012G091700 | Chr12 | 15,942,732 | 15,944,984 | 0.748 | 0.052 | 0.032 | 0.436 | C | CTP | plas |
| Gorai.012G097300 | Chr12 | 19,852,780 | 19,866,719 | 0.126 | 0.091 | 0.086 | 0.933 | _ | OTHER | plas |
| Gorai.012G174600 | Chr12 | 34,411,261 | 34,419,888 | 0.006 | 0.044 | 0.91 | 0.385 | S | SP | plas |
| Gorai.013G023900 | Chr13 | 1,748,031 | 1,750,061 | 0.212 | 0.073 | 0.042 | 0.876 | _ | OTHER | plas |
| Gorai.013G066600 | Chr13 | 7,566,031 | 7,570,626 | 0.025 | 0.108 | 0.305 | 0.89 | _ | SP | plas |
| Gorai.013G110000 | Chr13 | 24,930,003 | 24,937,157 | 0.184 | 0.316 | 0.045 | 0.566 | _ | OTHER | plas |
| Gorai.013G154700 | Chr13 | 42,608,245 | 42,614,083 | 0.101 | 0.053 | 0.365 | 0.181 | S | SP | plas |
| Gorai.013G154800 | Chr13 | 42,638,376 | 42,644,215 | 0.002 | 0.031 | 0.913 | 0.414 | S | SP | plas |
| Gorai.013G163700 | Chr13 | 44,488,785 | 44,493,251 | 0.003 | 0.103 | 0.168 | 0.935 | _ | OTHER | plas |
| Gorai.013G209700 | Chr13 | 52,142,788 | 52,146,665 | 0.033 | 0.073 | 0.113 | 0.912 | _ | SP | plas |
| Gorai.013G267600 | Chr13 | 57,999,151 | 58,006,969 | 0.179 | 0.1 | 0.129 | 0.8 | _ | OTHER | plas |
| Gorai.N013800 | scaffold_68 | 23 | 6,829 | 0.007 | 0.014 | 0.915 | 0.247 | S | SP | plas |

Note:Note: cTP: chloroplast transit peptide; mTP: mitochondrial targeting peptide; Other: nucleus, cytoplasmic or otherwise; SP: signal peptide; S: Secretory pathway; M: Mitochondrion; C: cytoplasm; -: other regions; plas: plasma membrane; cyto: cytoplasm; chlo: chloroplast; golg: golgi body; mito: mitochondrion and nucl: nuclear.
