# Supplementary material for: RNA-Sequencing, Physiological and RNAi Analyses Provide Insights into the Response Mechanism of the ABC-Mediated Resistance to Verticillium dahliae Infection in Cotton
Source: Genes (Basel). 2019 Feb 1;10(2):110. doi: 10.3390/genes10020110 (PMC6410047; doi:10.3390/genes10020110)
Supplement: Supplementary file 1 [file genes-10-00110-s001.zip › Supplementary files/Supplementary Table 4. Primers used in qRT-PCR analysis of ABC genes expression pattern..docx]

**Supplementary Table 4.** Primers used in qRT-PCR analysis of ABC genes expression pattern.

| Gene ID | Forward sequence (5'-3') | Reverse sequence (5'-3') |
| --- | --- | --- |
| Gorai.001G075900 | CCAAAATCAGGAAATAAGTG | TTAAGTGTTATTCCCTTGAG |
| Gorai.001G133500 | CTACTTCTACTAAACCCAAG | TGTTAGATTGAGTTTGTTCT |
| Gorai.001G182400 | GTTGGGGTGATGACGGAA | TGGATGCTAACAATGCGG |
| Gorai.001G254500 | TAATATCCACCATTTTCGTTT | TATCTCTCCTTACAGGATCA |
| Gorai.001G256100 | GGAAGAAGTATTTAGAAGCA | CAGTTGAAACACTAAAAACA |
| Gorai.001G258400 | GATCTTATAGCAGTTTTCCA | TTTTCTTTCTCTGTTTGTTG |
| Gorai.002G153900 | TATATTACCAGTGCTTTTCA | ATCGCTAATCCAAGACC |
| Gorai.002G162300 | ATTACAGTGAAACACAAAAC | TAGCAATGTAGTAAGATTCG |
| Gorai.002G246800 | GAGGATAGAACAATGGAAAT | TTCTTCTGTTTGAATTTTGG |
| Gorai.003G047600 | CTGTCATTACTGTTTCGC | TCCTTTACCCTTGCCTCG |
| Gorai.003G070300 | CCATGGTACTCTATTTTCTC | AGCTGGTTTAATATTTCCAT |
| Gorai.004G131200 | TGCGTTTCTGACTTTTCC | ACAGTTGGCTTCGGTTTA |
| Gorai.004G147200 | CTTCTTCTTTCTGTTTTCTG | AAGAAAACTTGGAAAACATC |
| Gorai.004G177600 | CATGATTTTATAATGGCGAC | GCAAAACTCACAGATAGG |
| Gorai.004G261400 | TCATCCGCTCTGTCCATA | CCAAACAACCTTCCCCTC |
| Gorai.005G221800 | AATGGGTCCTTCGGGTTG | TGTCCTGCTTGGCTTGTG |
| Gorai.006G021600 | TACATCGGAGCTGGTCTA | CGTGTTGTGAGGTTTTCT |
| Gorai.006G126900 | AAAACACAGTGGTGAAGCA | AACACAAATAGATAAAGCC |
| Gorai.006G157200 | AACCGATAGAGTGGGGAT | GAGAAGAGTAGGCAGGGC |
| Gorai.007G035900 | GTTAGTCATTTTGGATGAAC | CTTAAAACATCAGCTTCTTC |
| Gorai.007G234000 | ATTGGAAACACAATTTCATC | AGAGCAATAAGACAAAGTAG |
| Gorai.007G239200 | TTTTGAAGAAGAAATGGTTG | ATATAATAAGCTCAAACCCC |
| Gorai.007G244600 | TCCAATACAGAAGAAATTGA | ATAGCTCTTGGTAATGTTTT |
| Gorai.007G310600 | GGGAGAACAGGCAGTGGT | AGGGATGATGCTGAGTCG |
| Gorai.007G310700 | TCGTTTGTGTCTGTAGCC | TTGTGTCAGGAAGGTTGT |
| Gorai.007G310800 | TTTCGTTTTCTGGGTTGC | AAGGTTGTAGATTGGCTC |
| Gorai.008G047300 | TTCTCCAAGTGAAAAGATAG | AGTTCTACAATCCGAAATAG |
| Gorai.008G047500 | TTTTGATAAATGCGTAAAGG | AATTCTATCAACCTGTGAAA |
| Gorai.008G191700 | TGCAATGTTTTCAAATGTTA | CAGGAAATAACTCCATTACA |
| Gorai.008G219500 | GAATACTAATCAAGGAACGA | CCAATAGATTATGAAGACGA |
| Gorai.008G271700 | TTTTGTGGCTACCTTAAATA | GTCATATTCCAACACAGATA |
| Gorai.009G129000 | CTATTGAAGGGTATTAGTGG | ATCCATTAGAGTGGTTTTAC |
| Gorai.009G302000 | TATAATGCTCGAAGGAAAAA | GTTAAGGAAATCAGTCTTCA |
| Gorai.009G342400 | CCATTAACGCTTGTGCTC | AATCCTTTGCTTCTGCCC |
| Gorai.010G181700 | TCCTTGCCCCTCGTTTAC | TCGCTTTCTGGGTCCTTA |
| Gorai.011G066800 | TCCCAAAAACCAAGTCAC | CCATAAGCTGCAGTACCA |
| Gorai.011G071700 | CTTCCCTTCACTCCTCTA | AACCTATCCTCACCAACT |
| Gorai.011G166900 | GAGATTGCTGGATGGAGT | TTGAGGATTGTGGTGGTT |
| Gorai.011G205700 | GCTGTGGCTAATGGTGTG | GCTGAGTTCCTGAGGTGG |
| Gorai.011G220300 | AAGAAGAAAAGGAGAATGAC | GTTCATTAGGCTTCAAAATC |
| Gorai.011G295700 | AAATCTCATTAGCCATCTAC | CTGGATTCTTGTTATTGGTA |
| Gorai.012G034800 | ACCTCTCTGCTCCATCTA | TTTGCCTTGCCTCGTATT |
| Gorai.012G035000 | CTAATGGTTGTGGTAAATCT | GTCTGGATTTTGGAATACAT |
| Gorai.012G038700 | CTAATTCTGGAAGTGAAAGA | AAATACTCTCCAAAATCCAA |
